# Recent advances and challenges of rare variant association analysis in the biobank sequencing era

**DOI:** 10.3389/fgene.2022.1014947

**Published:** 2022-10-06

**Authors:** Wenan Chen, Brandon J. Coombes, Nicholas B. Larson

**Affiliations:** ^1^ Center for Applied Bioinformatics, St. Jude Children’s Research Hospital, Memphis, TN, United States; ^2^ Department of Quantitative Health Sciences, Mayo Clinic, Rochester, MN, United States

**Keywords:** rare variant, sequencing data, variant annotations, population structure, external controls, family-based design, complex phenotypes, case-control

## Abstract

Causal variants for rare genetic diseases are often rare in the general population. Rare variants may also contribute to common complex traits and can have much larger per-allele effect sizes than common variants, although power to detect these associations can be limited. Sequencing costs have steadily declined with technological advancements, making it feasible to adopt whole-exome and whole-genome profiling for large biobank-scale sample sizes. These large amounts of sequencing data provide both opportunities and challenges for rare-variant association analysis. Herein, we review the basic concepts of rare-variant analysis methods, the current state-of-the-art methods in utilizing variant annotations or external controls to improve the statistical power, and particular challenges facing rare variant analysis such as accounting for population structure, extremely unbalanced case-control design. We also review recent advances and challenges in rare variant analysis for familial sequencing data and for more complex phenotypes such as survival data. Finally, we discuss other potential directions for further methodology investigation.

## Introduction

High-throughput next-generation sequencing (NGS) technologies, including whole-exome sequencing (WES) and whole-genome sequencing (WGS), are increasingly being applied in studies of both rare diseases and common complex traits. In contrast to the array-based genotyping commonly applied in genome-wide association studies (GWAS), WES/WGS can directly capture relevant variation not interrogated by common genotyping platform designs, including rare variants (RVs). Identifying rare variants is important because pathogenic rare germline mutations can cause many human diseases. For example, many SOD1 mutations can cause amyotrophic lateral sclerosis (ALS) (Sau et al., 2007), NF1 mutations can cause pediatric brain tumors (Campian and Gutmann, 2017), RB1 mutations can cause retinoblastoma (Yun et al., 2011), and ETV6 mutations can cause pediatric acute lymphoblastic leukemia (Hock and Shimamura, 2017). For adult cancers, mutations in BRCA1/BRCA2 can cause breast and ovarian cancer (Mavaddat et al., 2013), mutations in TP53 are responsible for many pediatric or adult cancers or syndromes (Olivier et al., 2010). Mutations in APP, PSEN1, PSNE2 can increase the risk of early onset Alzheimer disease (Lanoiselee et al., 2017). Therefore, sequencing technologies have often been prioritized for studying both somatic and germline DNA mutations in cancers (Consortium, 2020), and germline pathogenic mutations in rare Mendelian diseases (Gilissen et al., 2011).

There is also increasing interest in exploring the contributions of RVs to variability in common complex traits, driven in large part by the phenomenon of “missing heritability” (Manolio et al., 2009). This missing heritability is defined by the commonly observed gap between complex trait heritability estimates from family-based studies and trait variation explained by common single-nucleotide polymorphisms (SNPs) discovered by large-scale GWAS, leading to the common-disease/rare-variant (CD-RV) hypothesis (Schork et al., 2009). The CD-RV genetic model postulates that common complex traits may be the result of multiple RVs that impact one or multiple genes that would not be tagged by conventional GWAS SNPs. RVs have also largely remained unexplored in the GWAS era of genetic association analysis, and the vast majority of human genetic variation is rare. Technology and sample sizes have started to bear this hypothesis out, as RVs have recently been shown to account for unexplained heritability in highly polygenic traits, such as height and BMI (Wainschtein et al., 2022). Given the increasing empirical evidence that RVs play a role in various complex traits, cancers and rare diseases, such as results from WES profiling of the United Kingdom Biobank (Wang et al., 2021), NGS is increasingly being used to investigate RV associations in risk of human disease.

Unlike common variants (CVs), application of traditional single-variant analysis methods on RVs is often underpowered for typical NGS study sample sizes due to low minor allele frequencies (MAFs). The multiple testing burden for single RV analysis also increases as a function of sample size due to the fact that more unique RV positions will be detected. Consequently, adequate power for single-variant RV analyses requires extremely large sample sizes that often are practically and/or economically unfeasible. Moreover, it is possible *via* allelic heterogeneity that multiple RVs within a gene may affect the same trait. Therefore, RV analysis using NGS data is typically performed using “aggregative” testing, whereby identified variants are tested collectively in some fashion based on physical overlap with pre-defined genomic regions. Table 1 shows a comparison between CV and RV association analysis.

**TABLE 1:** Comparison between CV and RV association analysis.

Considerations	CV association analysis	RV association analysis
Assays	Typically captured using inexpensive genotyping microarrays	Often requires NGS, especially for detecting extremely rare/novel variants
Number of variants tested	Often single variant based (e.g., GWAS)	Often multiple variants based due to low power of single-variant methods
Population structure	Confounding can be adequately controlled using PCA or mixed models	Rare variants are likely more recent and reflect finer subpopulations. May need either more PCs or specifically designed methods
Null distributions of test statistics	Ordinary asymptotic distributions work well	Null distributions are often complex mixtures and more sophisticated methods may be necessary
Use of annotations	Statistical test for each variant is often performed without relying on annotations	Due to the large number of rare variants in a region, annotations are often used to filter rare variants
Interpretation	Due to potential LD, single-variant associations may be tag-SNPs	May be unclear which RVs are “driving” a significant RV association result using aggregative testing, especially those considering both directions

In this review, we discuss emerging challenges and methodological advancements in RV association analysis, covering topics related to variant filtering and annotation, population structure, implications of study design and use of externally-sequenced control samples, and adaptation of existing methods to different phenotypes. With the growing availability of DNA sequencing datasets with sufficiently large sample sizes for well-powered RV association analyses, the content of this review is particularly topical as investigators focus their attention on the role of RVs in human traits.

## Background on RV association testing methodology

While many RV testing methods have been available for over a decade, they may still largely be considered niche even among genetic epidemiologists given the only recent emergence of DNA sequencing datasets with sufficiently large sample sizes. In this section, we briefly review a basic background of RV association analysis, orienting the reader to core concepts that contextualize modern methodological challenges and advancements.

### What is “rare”?

No formal threshold is defined for what qualifies a variant as an RV. For GWAS, minimum MAF thresholds are often applied to exclude SNPs that are underpowered for single-variant association analysis - typically in the range of 0.5%–5.0%, depending on available sample size. Current convention partitions variants into ultra-rare, rare, low-frequency, and common, with respective population MAF thresholds of 0.05%, 1% and 5% often observed in the literature. For RV association testing, this definition is more readily important, as it defines which variants are eligible for analysis. While this threshold is left to the investigator, 1% and 5% thresholds are commonly applied in practice for common complex traits, while even lower MAF (e.g., 0.1% or 0.05%) have been used for cancer predisposition variants or rare Mendelian diseases.

### Defining variant sets

**FIGURE 1:**
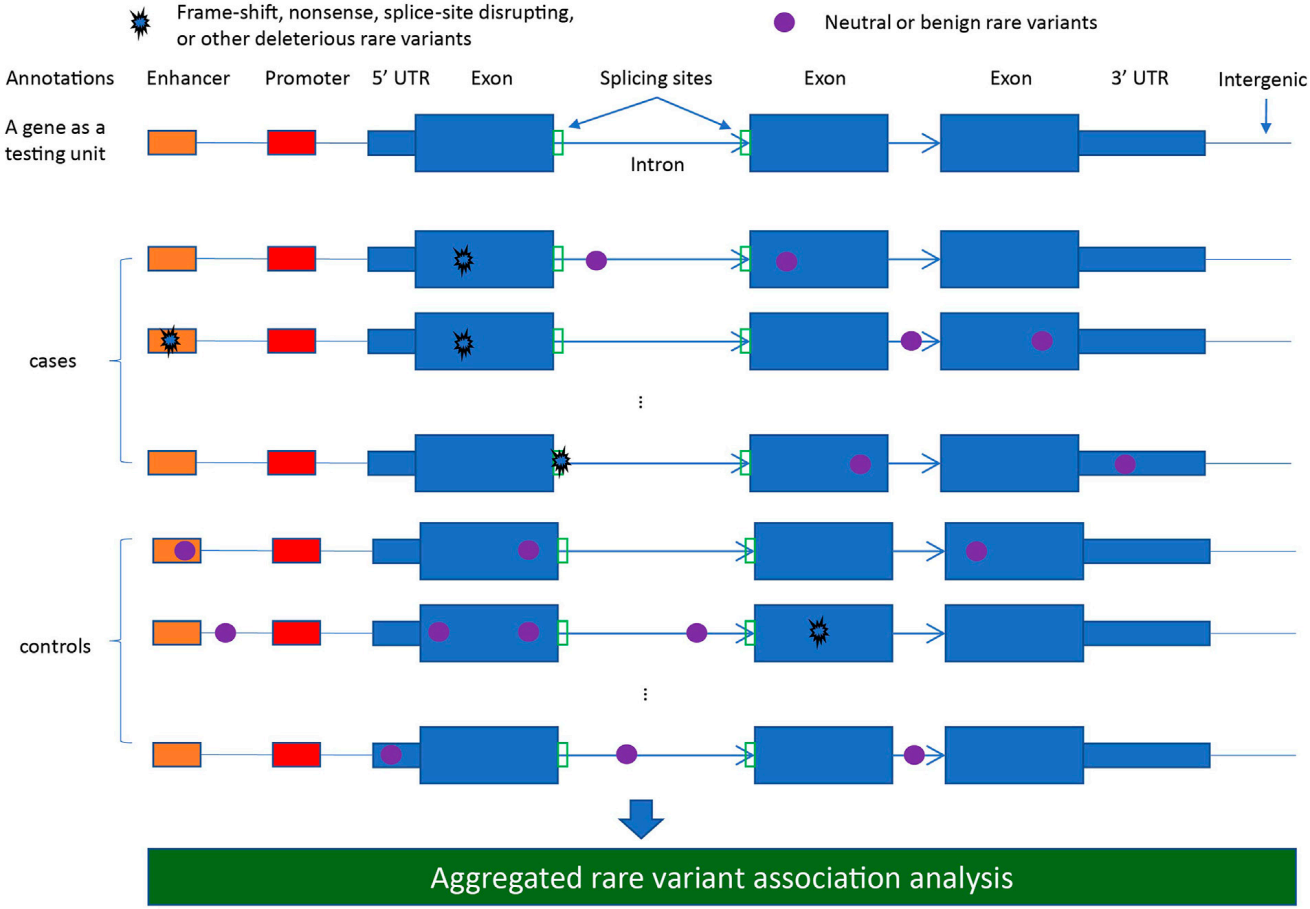
A diagram illustrating different rare variant types defined from annotations for aggregated rare variant association analysis.

Conducting aggregative testing naturally requires defining eligible variant sets for analysis, which generally is akin to defining genomic region(s) by which overlapping RVs are grouped. Such regions should be defined *a priori*, as they 1) enumerate the anticipated multiple testing burden and 2) prevent overfitting *via* selection of genomic regions that correspond to chance RV enrichment. The most commonly applied region-based testing unit is a gene (Figure 1), particularly for large-scale agnostic scans (e.g., WES/WGS). More focused candidate gene studies may examine a finer regional granularity, such as individual exons or protein functional domains. Alternative approaches to standard region-based testing include scan-type statistics (Ionita-Laza et al., 2012; Schaid et al., 2013b), where the testing unit is a sliding genomic window, and pathway/gene-set testing (Wu and Zhi, 2013), where gene-level results may be further combined across biologically-related sets of genes.

### Types of RV tests

Many aggregative RV analysis methods have been proposed in the literature, with the majority falling into two broad classes: 1) burden tests and 2) variance-component, or “kernel”, tests. For the latter, the set-based Sequence Kernel Association Test (SKAT) (Wu et al., 2011) and its variations (e.g., SKAT-O (Lee et al., 2012)) are most widely applied, although other competing approaches and modifications have been developed.

First, we must define some relevant notation for RV testing. Specifically, let us consider a sequencing-based genetic association study of N samples on some phenotype of interest, defined by vector $Y_{N\times 1}$ . For our purposes, we assume Y to be continuous or binary in nature, as these phenotype classes are broadly supported by most statistical methods for RV association analysis. We define available genotype allelic dosage data on M identified variants, $G_{N\times M}$ , such that $G_{ij}\in \left\{0,1,2\right\}$ . Some methods also allow for covariate adjustment (e.g., age and sex), and we define the set of P additional adjusting covariates by the matrix $X_{N\times P}$ .

The first class of RV tests is a burden test. Generally, a burden test generates a test statistic based upon a (potentially weighted) sum of observed RVs, which implicitly assumes that causal variants share effect directionality (e.g., benign vs deleterious). The RV burden for subject i may then be calculated as $B_{i\;}=\overset{M}{\underset{j=1}{\sum }}w_{j}G_{i,j}$ , where optional variant weights are defined as $w={\left(w_{1},\ldots ,w_{M}\right)}^{\prime }$ . These weights should be defined to reflect relative confidence in causal status and/or anticipated magnitude of effect on the phenotype of interest, and well-informed weight definitions can substantially impact analysis results. One of the simplest burden testing procedures is the collapsing and sum test (CAST) (Morgenthaler and Thilly, 2007), which is a 2 × 2 Fisher’s Exact Test for a binary RV carrier status for case-control studies. In this test, burden is further reduced to an indicator variable $B_{i}^{*}=I\left(B_{i}>0\right)$ and samples are classified in the contingency table by burden status. The concept of variant burden has been generalized to a large number of testing frameworks where a univariate exposure is compared to an outcome of interest (e.g., combined multivariate and collapsing test, weighted-sum statistic test). Burden measures can also be used as predictors in regression analysis if additional covariate adjustment is desired. Adaptive burden testing approaches were developed to incorporate data-driven approaches to weighting and filtering of variants, including the variable threshold test (Price et al., 2010) (Han and Pan, 2010). Many of these adaptive burden tests employed permutations to compute *p*-values, which can be computationally burdensome. The burden test can also be framed as a score test to derive analytical *p*-values, such that the statistic $Q_{B}=(\overset{N}{\underset{i=1}{\sum }}\left(Y_{i\;}-{\hat{Y}}_{i\;}\right)B_{i}{)}^{2}$ follows a scaled $\chi_{1}^{2}$ distribution, where ${\hat{Y}}_{i}$ is the predicted value of $Y_{i}$ under a null model $Y_{i\;}=\beta_{0}+X_{i}\beta +\epsilon_{i}$ , X represents non-genetic variables with effects β , and the genetic effects corresponding to G are all fixed at zero.

In contrast to burden tests, kernel tests are robust to the presence of non-causal variation and heterogeneity of effect directionality. These tests are based upon measures of genetic similarity in the form of a kernel matrix $K_{N\times N}$ , where $K_{i,j}=\kappa \left(G_{i},G_{j}\right)$ for some kernel function κ(⋅,⋅) describing the similarity between genotype vector $G_{i}$ of subject i and genotype vector $G_{j}$ of subject j . A common kernel function is the weighted linear kernel, such that $K=GWWG^{\prime }$ , where $W=diag\left(\sqrt{w_{1}},\ldots ,\sqrt{w_{M}}\right)$ for the vector of marker weights w . The score test statistic is then given by the equation $Q={\left(Y-\hat{Y}\right)}^{\prime }K\left(Y-\hat{Y}\right)$ where $\hat{Y}$ is the predicted value of Y under the null model. The null distribution for Q then follows a mixture of $\chi^{2}$ distributions, which can be well-approximated by a variety of methods or exactly computed.

SKAT was later extended to a generalized framework that includes formulation of a kernel function for the burden test score statistic, $Q_{B}$ . SKAT-O, aka “Optimal SKAT” (Lee et al., 2012), is a type of hybrid approach to RV testing that optimally combines both burden and kernel statistics, $Q_{B}$ and $Q_{S}$ , respectively, into a weighted average, such that $Q_{\rho }=\rho Q_{B}+\left(1-\rho \right)Q_{S}$ . Selection of ρ is conducted by SKAT-O using a simple grid search over the unit interval. Also known as omnibus tests, methods like SKAT-O are data-adaptive and consider a broad spectrum of potential genetic architectures rather than selecting one over the other. In general, there is no uniformly most powerful test across all potential conditions, since factors such as magnitude and direction of effect sizes, relationships between effect size and MAF, and proportion of causal variation all influence the relative power for a given test. While the robust property of kernel tests has great appeal, a burden test will be more powerful under conditions of high causal variant proportion. For large agnostic scans (e.g., WES/WGS studies), flexible omnibus tests like SKAT-O are often recommended.

Many other RV methods have been proposed that are neither burden tests nor variance component tests, such as the replication-based test (RBT) or *p*-value combination methods. The RBT instead tests for enrichment of rare alleles in cases and controls (Ionita-Laza et al., 2011). Alternatively, *p*-value combination methods combine the group of RV *p*-values in a given gene using either Fisher-like Method such as Fisher (Derkach et al., 2013), TFisher (Zhang et al., 2020), GFisher (Zhang and Wu, 2022), or some other transformation. These methods include the Aggregated Cauchy Association Test (ACAT) (Liu et al., 2019) which transforms *p*-values to the Cauchy distribution, the Higher Criticism (HC) or generalized HC test which combines ordered *p*-values using the HC statistic (Xuan et al., 2014) (Barnett et al., 2017), and the Generalized Berk-Jones (GBJ) test (Liu et al., 2021).

## Recent advances and challenges in RV association analysis

RV association studies present a number of unique challenges that have driven methodological development in the last decade; however, many challenges remain outstanding. We summarize our review of recent advances and challenges of RV association analysis in Table 2. The essential themes in these topics align with fundamentals of hypothesis testing: type I error control, maximizing the statistical power, and how to model different data types in a statistical test. For example, accounting for population structure and extremely unbalanced case-control designs address the challenge of inflated type I error in RV association tests. Incorporating variant annotations and using external controls aim to increase the statistical power of RV association tests. Analysis of familial sequencing data needs to model the inheritance patterns of genotypes and genotype correlations among family members, treating related samples as unrelated will lead to inflated type I error. Analysis with more complex phenotypes requires modeling the additional complexities in phenotypes in order to achieve well controlled type I error and powerful test results. We provide a more detailed review of each topic in the following sections.

**TABLE 2:** Outline of advances and challenges of RV association analysis.

Topic	Motivation/Challenges
Incorporating variant annotations	There is growing knowledge available on potential variant impact on protein structure and function, and annotations may provide useful information in selecting functional variants. However, relevant annotation may vary by gene and phenotype, and annotation-informed filtering/weighting of variants may lead to improved or decreased statistical power
Accounting for population structure	Population structure is a primary confounding factor in genetic association analysis, and properly controlling for these confounding effects may differ relative to common variants
Accounting for extremely unbalanced case-control designs	Large biobanks with rare outcomes have led to extremely unbalanced case-control designs. This inflates type I error of standard RV methods relying on large sample theory based asymptotic distributions
Increasing power using external controls	To reduce the sequencing cost, often only cases and few controls are sequenced. In order to perform RV association analysis, external controls are used. One main challenge of this design is the potential confounding batch effect from different sequencing and processing platforms between cases and controls
Analysis of familial sequencing data	Family based design has the advantage of being robust from population structure, it is also the standard way for heritability estimation. It is important that RV association analysis methods can accommodate studies using the family based design
Allowing for more complex phenotypes	While case-control studies and analyses of quantitative traits are most common in RV analysis, RV methods have also been developed for multivariate phenotypes and time-to-event outcomes

### Incorporating variant annotations in RV analysis

The statistical power of most aggregative RV testing methods is highly dependent on the proportion of truly causal variants included in the RV set. Given that the functional relevance status of individual variants is generally not known *a priori*, variant filtering and/or weighting is common practice to leverage biological knowledge and improve power, and many RV testing methods are designed to flexibly accommodate variant weights in the testing procedure. For burden tests, it has been shown that the optimal weights will be proportional to the true absolute variant effect sizes (King and Nicolae, 2014). Absent any relevant functional annotation, weighting schemes based on MAF, such as the Madsen-Browning weights (Madsen and Browning, 2009) or beta density function weights (Wu et al., 2011), are commonly employed. This is motivated by an assumed inverse relationship between allele frequency and functional impact imposed by strong purifying selection pressure on highly damaging variants.

For gene-based RV testing, the simplest strategies incorporating annotation involve variant filtering based on the likely functional impact on the resulting protein product. Standard bioinformatics annotation tools (Wang et al., 2010) (Cingolani et al., 2012) (Mclaren et al., 2016) can rapidly assign basic qualitative functional variant effects based on the open reading frame of protein-coding gene transcript(s), and prioritization of loss-of-function variants (i.e., nonsense, splice-site disrupting, frame-shift indels) is commonly applied given the severity of the effects on the resultant protein structure. Variants that impose more modest changes to the amino acid sequence (i.e., missense, in-frame indels) may be more likely tolerated in relation to protein function, and a vast array of functional impact prediction tools have been developed to provide quantitative functional prediction scores to reflect the likelihood of deleteriousness (Livesey and Marsh, 2020). Synonymous and non-coding RVs may also impact a given gene through other mechanisms beyond direct alteration of the amino acid sequence, including disruption of regulatory sequences as well as epigenomic impacts. Many such annotations may also be cell-type specific, requiring consideration for the phenotype under study. Appropriate consideration for variant filtering and weighting may substantially improve statistical power for RV association discovery (Byrnes et al., 2013); conversely, misspecification of variant weights could lead to loss of power by inadvertently removing and/or down-weighting key disease-related functional RVs (Minica et al., 2017).

Given the number and heterogeneity of available variant annotations along with the uncertainty as to which annotations are most relevant to a particular gene-phenotype relationship, various methods have recently been proposed to dynamically accommodate and combine multiple annotations. For example, Wu et al. (Wu et al., 2013) proposed a multi-kernel approach using perturbation to perform kernel-based testing while simultaneously considering multiple candidate kernels, which could be defined by various competing weighting schemes. Due to the computational considerations of permutation/perturbation-based strategies, He et al. (He et al., 2017a) proposed the functional score test (FST), which similarly accommodates multiple candidate variant weighting schemes by partitioning the overall genetic effect attributable to the various annotation sources. The authors then apply a minP approach for combining test results across weight sets, and derive a computationally efficient resampling-based procedure for *p*-value calculation. More recently, Li et al. (2020) developed STAAR, which applies principal components analysis to matrices of various candidate annotation classes in order to reduce the annotation dimensionality. For gene-based testing, STAAR also considers testing stratified by variant classes, and all tests are then combined under an omnibus using the ACAT method.

### Accounting for population structure in RV analysis

The primary confounding factor in genetic association analysis of both common SNPs and RVs is population stratification, which is the systematic difference in allele frequencies across sub-populations due to non-random mating and genetic drift. Various statistical methods have been successfully developed to address confounding by population stratification for common SNP association testing in genome-wide association studies. The most popular of these approaches include principal component analysis (PCA) (Price et al., 2006) and (generalized) linear mixed models (GLMMs) (Kang et al., 2010). PCA-based methods often address population stratification by adjusting for the leading PCs derived from the genotype-dosage matrix as covariates in a regression-based analysis. In contrast, GLMMs can simultaneously account for population stratification and cryptic relatedness by modeling a random effect whose covariance structure is defined by an estimated genetic relatedness matrix (GRM).

Since most modern RV association testing methods are also regression-based, both PC adjustment and GLMM-based strategies can be readily accommodated to address population stratification in RV analyses. However, it has been less clear whether the same methods applied for common SNPs can be similarly effective for RV association testing. From a population genetics perspective, it has been argued that RV associations are more prone to confounding effects of population stratification, as RVs are likely to be more recent and thus will reflect finer population substructure (e.g., regional geographic differences) (Mcclellan and King, 2010) (O’connor et al., 2015). To this end, a larger number of leading PCs could be required when performing RV testing to account for more nuanced population stratification (Mathieson and Mcvean, 2012). However, it has been shown that this may not be sufficient, as additional PCs derived from common SNPs may not capture fine-scale population stratification (Persyn et al., 2018). This is commensurate with other findings that demonstrate that common and RVs can reflect systematically different patterns of structure (Mathieson and Mcvean, 2012; Ma and Shi, 2020). Similarly, substantially different PCs may be obtained when derived from genotype matrices that are composed of common variants, RVs, and both (Liu et al., 2013; Ma and Shi, 2020).

Given the uncertainty as to how to properly account for population stratification in a regression-based analysis framework for RVs, alternative strategies based on sample matching have also been proposed. Matching based on genetic ancestry typically involves the use of leading PCs and makes less assumptions about the functional relationship of the PCs confounding the association between RV genotypes and outcome. Cheng et al. (2022) proposed a family of RV tests based on conditional logistic regression (CLoMAT), along with a matching algorithm based on PCA output. Another recently developed method used local permutations (LocPerm) to account for the population structure in the association test (Bouaziz et al., 2021; Mullaert et al., 2021). LocPerm first defines the K-nearest neighborhoods of each sample based on top PCs calculated from common variants. Then it selects permutations such that each phenotype is drawn from the K-nearest neighbors. Simulation results by the authors showed that LocPerm can control type I error rates under a variety of study conditions. However, the permutation procedure may require high computation cost when the sample size becomes large.

### Accounting for extremely unbalanced case control design in RV analysis

The decrease in sequencing costs and the increase in large biobanks established around the world now enable researchers to identify the role of RVs in complex and sometimes rare outcomes (Backman et al., 2021). Many of these samples contain rich phenotypic data through surveys and questionnaires as well as linking to the electronic health record, which allows for investigation of RV associations phenome-wide. Barring any concerns of selection bias, it is generally optimal under these study conditions to include all genotyped samples in an association analysis. Since most diseases have a low prevalence in these biobanks, this leads to association tests with extremely unbalanced case-control samples. Many of the single RV and multiple RV tests mentioned above, such as SKAT and weighted versions of SKAT, take advantage of the score test framework to dramatically increase computational efficiency of RV tests by avoiding calculation of the likelihood or maximum-likelihood estimator under the full model. In the case of severe imbalance, violation of the large sample theory assumptions used to derive the asymptotic distribution leads to inflated type I error rates of the score test (Zhang et al., 2019). Recent methods have addressed this by applying either Firth regression (Wang, 2014) or a saddle-point approximation (SPA) (Zhou et al., 2018) to both single RV and multiple RV tests.

Firth regression uses a penalized likelihood approach to remove bias from the maximum-likelihood estimates. As the sample size increases, this penalization shrinks to zero; however, in the instance of extreme imbalance, this term helps maintain control of the type I error rate (Wang, 2014). A limitation of this approach involves requiring the calculation of the maximum likelihood under both the null and the full model for a likelihood ratio test, which is computationally expensive in large biobank-scale datasets and becomes impractical when considering RV testing across the genome. Alternatively, instead of assuming a normal approximation for the score test, application of SPA estimates the null distribution using all the cumulants hence all the moments in the case of severe imbalance and controls the type I error rates well (Dey et al., 2017).

The SPA approach is implemented in SAIGE (Zhou et al., 2018) and in REGENIE (Mbatchou et al., 2021) for testing single-variant association across the genome in the case of extreme imbalance. The SPA approach has also been used to extend SKAT and SKAT-O testing of multiple RVs and avoid the inflated type I error of those tests in the case of severe case-control imbalance (Zhao et al., 2020). REGENIE also alternatively implements approximate Firth regression to allow for usable SNP effect sizes because the SPA approach can sometimes fail to produce good estimates of SNP effect sizes and standard errors. A comparison of these methods in the United Kingdom Biobank testing for association in rare diseases found that SAIGE and REGENIE (SPA and Firth) appropriately controlled the type I error, but the SAIGE and REGENIE-SPA had inflated effect-size estimates (Mbatchou et al., 2021). Furthermore, REGENIE was 4.4 times faster than SAIGE in terms of CPU time (Mbatchou et al., 2021). Finally, the SPA approach has also been implemented in SPAGE to allow for scalable genome-wide single-variant gene-environment interaction analyses, which are well calibrated for severe case-control imbalance (Bi et al., 2019).

### Using external controls in RV analysis

Because RV analysis often requires tens of thousands of samples to reach adequate statistical power, using available external sequencing data as a source of controls is a cost-effective approach for case-control RV association studies (Wojcik et al., 2022). One major challenge of using external controls is the potential confounding batch effect due to different sequencing platforms and genotype calling bioinformatics pipelines. For example, the sequencing depth between cases and controls can vary considerably if cases are WES samples (average depth 80x) and controls are low read depth WGS samples from the 1,000 Genomes Project (average depth 7x) (Genomes Project et al., 2015).

Several computational methods have been developed to address these challenges (Table 3). When individual sequencing data are available, statistical models have been developed to incorporate the read depth or genotype likelihood into the association test. Derkach et al. (2014) developed a score statistic that uses the expected genotype instead of the called genotype to account for the differences in read depth. Hu et al. (2016) developed a likelihood-based approach incorporating the sequencing reads depth directly without calling the genotypes; however, due to the direct use of raw sequencing reads, the computational cost might be high. Chen and Lin (2020) proposed regression calibration (RC)-based and maximum likelihood (ML)-based methods to incorporate the genotype likelihood in the association test and also allow inclusion of covariates to adjust for confounding, such as population structure. When internal controls are available, Li and Lee (2021) developed a weighted sum of score statistics to allow inclusion of both the internal and external controls by assessing the existence of batch effects between the internal and external controls for each variant.

**TABLE 3:** Summary of methods using external controls for improvement of statistical power.

Method	External control data	Require internal control?	Require sequencing depth for cases and controls?	Method correcting for batch differences between case controls	Can the method adjust for covariates?	Test
RVS (Derkach et al., 2014)	Individual genotype likelihood	N	N	Modeling the effect of sequencing depth	N	Single variant based test, burden test and variance component based test
TASER (Hu et al., 2016)	Individual Bam files	N	N	Modeling the effect of sequencing depth	N	Burden test
Chen and Lin (Chen and Lin, 2020)	Individual genotype likelihood	N	N	Modeling the effect of sequencing depth	Y	Single common variant based test
iECAT-Score (Li and Lee, 2021)	Individual genotypes	Y	N	Only use the external control if no batch effect exists	Y	Single variant based test for common and rare
iECAT-O (Lee et al., 2017)	Summary counts	Y	N	Only use the external control if no batch effect exists	N	A combination of burden test and variance component based test
ProxECAT (Hendricks et al., 2018)	Summary counts	N	N	Use non-functional variants as a baseline in the test	N	Burden test based on rare allele counts
TRAPD (Guo et al., 2018)	Summary counts	N	≥ 10 in 90% of samples	Adjusting filtering criteria	N	Burden test based on sample counts
RV- EXCALIBER (Lali et al., 2021)	Summary counts	Preferred	≥ 20 in 90% of samples	Adjust the expected counts sample-wise and gene-wise	N	Burden test based on rare allele counts
CoCoRV (Chen et al., 2022)	Summary counts	N	≥10 in 90% of samples	Consistent filtering to keep high quality variants	N	Burden test based on sample counts

Methods have also been developed using publicly available summary genotype counts of external controls, such as gnomAD (Karczewski et al., 2020). Since summary counts have less information than individual sequencing data, it is even more challenging to correct for batch effects between cases and external controls. When both internal controls and external summary counts are available, Lee et al. developed a method iECAT-O (Lee et al., 2017) that can use external summary counts when batch effects between internal and external controls cannot be detected. There are other methods developed that do not assume the existence of internal controls and aim to adjust for the batch effects between cases and external controls. ProxECAT (Hendricks et al., 2018) assumes the non-functional variants within a gene can be used as a proxy of how the variants are sequenced and called. The total number of rare alleles from functional variants and non-functional variants are then compared between cases and controls. TRAPD (Guo et al., 2018) uses coverage summary statistics to keep high quality positions and then uses synonymous variants to tune variant filtering parameters between cases and controls. A burden test is used assuming RVs are independent from each other and thus can be pooled together from summary counts of individual variants. RV-EXCALIBER (Lali et al., 2021) also uses coverage summary statistics to keep high quality positions, instead of using the raw summary counts from public controls, it adjusts them using gene-wise and sample-wise correction factors and then compares the corrected values from public controls with observed values in cases. In addition to using coverage summary statistics to filter variants, a recently developed method CoCoRV (Chen et al., 2022) can provide consistent filtering between cases and controls. It also uses a blacklist to filter out potential problematic variants that show large discrepancies between the WES and WGS cohort. CoCoRV also provides a way to handle RVs in high linkage disequilibrium (LD) and can perform ethnicity-stratified association analysis which ameliorates potential confounding due to population structure.

A notable limitation of methods using summary counts is that they cannot adjust for covariates, given that only the summary information is available for controls. Therefore, adjusting for the confounding due to population structure in these methods remains challenging. Careful matching of race/ethnicity between cases and controls is critical in these analyses. Given that high-coverage WES (∼80x) and WGS (∼30x) external control data are becoming more and more common, evaluating the performance of methods modeling sequencing depth directly or using simple read-depth based filtering criterion would provide guidance on how to combine sequencing data sets in association tests.

### RV analysis of familial sequencing data

Familial or pedigree-based design has the advantage of being robust to population stratification when using proper analysis methods. It is also indispensable if the interest is to study the effect of pathogenic *de novo* variation on risk of the disease. In addition, pedigree data from previous linkage mapping efforts might be sequenced for additional analysis (Ott et al., 2015). Recent advances in RV association analysis for pedigree data in general can be summarized into two categories. The first category includes methods developed to analyze RVs based on the transmission disequilibrium test (TDT) or family-based association test (FBAT) (Laird and Lange, 2006). The second category includes the association test methods that adjust for relatedness and population structure using mixed models.

RV association analysis for unrelated individuals has been introduced to FBAT, which is robust to the presence of population structure. For example, the burden test was introduced to FBAT by De et al. (2013). Ionita-Laza later introduced the SKAT-type test to FBAT (Ionita-Laza et al., 2013) and showed that the statistical power for dichotomous traits was comparable between a family-based study for 500 trios and population-based study of 500 cases and 500 controls. Hecker et al. (2020) recently proposed a general framework for RV association tests including the burden test, SKAT-type test, and higher criticism based test, which was more powerful when the signal was sparse. By combining the *p*-values from different RV association tests using ACAT (Liu et al., 2019), Hecker et al. (2020) demonstrated the proposed method had robust and more powerful performance than other TDT extensions, such as RV-TDT (He et al., 2014), RV-GDT (He et al., 2017b), and gTDT (Chen et al., 2015). Under the FBAT model, the phenotype is treated as fixed and the genotypes as random variables. Because FBAT conditions on the phenotype, it is robust to different ascertainment schemes based on phenotypes, such as selecting pedigrees enriched with cases (Schaid et al., 2013a; Hecker et al., 2019). One disadvantage of FBAT is that it conditions on the parental genotypes and does not use between-family information (Schaid et al., 2013a; Ionita-Laza et al., 2013), which can result in loss of power compared with the association tests adjusting for relatedness using regression models.

The second category of association methods account for the relatedness in a regression model. Schifano et al. (2012) and Chen et al. (2013) developed similar RV association tests for a quantitative trait using a linear mixed model. These methods extend the SKAT method to handle pedigree data by including a random variable to account for the correlation between individuals within the same pedigree. The correlation matrix between individuals within a pedigree can be defined using twice the kinship coefficient (Sinnwell et al., 2014). If the pedigree information is not explicitly available, often the GRM estimated using genome-wide common variants is used. For binary traits, the logistic mixed model approach GMMAT was developed by Chen et al. (2016). To account for unbalanced case-control ratios using the saddlepoint approximation and efficient resampling as used in SAIGE (Zhou et al., 2018), Zhou et al. developed SAIGE-GENE (Zhou et al., 2020) using the generalized linear mixed model which can handle both binary and quantitative traits. For the mixed model methods, they regard the genotype as fixed and the phenotype as random. The relatedness within each pedigree is then included in the covariance matrix of the phenotype. Besides the mixed models, two similar retrospective likelihood-based methods, PedGene (Schaid et al., 2013a) and FARVAT (Choi et al., 2014) were also developed. As in FBAT, both methods treat the phenotype as fixed, and the genotype as random variables. The covariance matrix of genotypes incorporates both the LD information and the pedigree information, and a score statistic is derived. Power evaluations have shown that for quantitative traits, based on a recent review (Choi et al., 2014) (Larson et al., 2019), PedGene had similar power to the mixed model based methods developed by Schifano et al. (2012) and Chen et al. (2013). In addition to burden and SKAT-like tests, a robust SKAT-O-like method was also developed in FARVAT. FARVAT was written in C++ and has a speed advantage over PedGene. Evaluations (Wang et al., 2016; Fernandez et al., 2018) have shown that PedGene and FARVAT are usually more powerful than TDT based methods such as RV-TDT (He et al., 2014) or RV-GDT (He et al., 2017b). Even though the regression model based methods that account for the relatedness are likely more powerful than TDT based methods, how well they can account for the population structure might need further investigation (Mathieson and Mcvean, 2012).

For RV association analysis using pedigree data, because the two categories of methods have their own advantages and potential disadvantages, it might be a good idea to try methods in both categories and summarize their results for a robust interpretation of the data.

### Allowing for more complex phenotypes in RV analysis

Many RV tests were developed to accommodate single binary and/or continuous outcomes. However, a given study may collect multiple and potentially highly related outcome measures. One extension of the above described methods is to consider these multiple correlated outcomes in order to increase statistical power and reveal potential pleiotropy. As is the case for testing association of multiple RVs with a single phenotype, testing for association of RVs with a multivariate outcome primarily uses either burden-like (Zhao and Thalamuthu, 2011; Zhu et al., 2015; Kaakinen et al., 2017) or SKAT-like (Ray et al., 2016; Liu and Lin, 2018; Dutta et al., 2019; Liu and Lin, 2019; Luo et al., 2020) approaches. Additional methods used are a standard MANOVA approach (Ferreira and Purcell, 2009) and a regression approach that flips the outcomes and RV predictor using proportional odds regression (MultiPhen) to test for association of a group of phenotypes with the RV as an outcome (O’reilly et al., 2012). However, no test among these is uniformly most powerful and many of these methods are sensitive to deviations from normality in the case of multivariate quantitative phenotypes (Ray and Chatterjee, 2020).

Another type of outcome that is especially common to biobanks is time-to-event data. Cox proportional hazards (PH) regression models are heavily used in this context, but fitting the maximum partial likelihood for these models is often not scalable to large GWAS. For that reason, kernel statistics using martingale residuals in place of residuals from a generalized linear model (e.g. SKAT) have been initially proposed for gene- or region-based RV testing across the genome (Chen et al., 2014; Larson et al., 2019), such as the method implemented in rareSurvival software (Syed et al., 2021). In the case of extremely unbalanced case-control designs, SPACox has been proposed to correct the inflated type I error rates in GWAS of RVs (Bi et al., 2020). This approach scales well by first fitting a Cox PH regression model only once across the genome-wide analysis and then using the SPA approach to calibrate the score statistics.

## Discussion

In this review, we have covered the basic background on RV association testing using sequencing data, and outlined leading areas of methodological development in RV association analysis. The growth in availability of large datasets with RVs measured will finally allow researchers to assess the impact that RVs have on rare and common diseases. This growing availability of large sequencing data not only makes RV analyses feasible, but may yield novel analytical issues. For example, many analytical issues may occur when trying to coordinate RV analyses across multi-site/biobank studies where incorporating all datasets into one conglomerated analysis is near impossible due to data sharing concerns and patient privacy. This means that RV analyses will likely require federated analyses with each site performing the analysis at their respective site for which results are combined afterward. Given the large number of potential rare variants that may be involved in a significant result, questions also remain as to how to optimally validate rare variant findings and how to design large-scale functional validation assays of the findings. Regardless of these potential challenges, the methodological advancements we have highlighted in this review demonstrate a very active scientific community dedicated to tackling these issues.

## Nomenclature

GLMM: Generalized linear mixed model
GRM: Genetic relatedness matrix
GWAS: Genome-wide association study
PCA: Principal components analysis
RV: Rare variant
CV: Common variant
SNP: Single-nucleotide polymorphism
